# Analysis of the population genetic structure and demographic history of *Tilia amurensis* and *Tilia japonica* in China using SSR markers

**DOI:** 10.3389/fpls.2025.1651814

**Published:** 2025-12-11

**Authors:** Yue Zhang, Fengqi Zang, Dan Liu, Yan Ma, Yizeng Lu, Qichao Wu, Dekui Zang

**Affiliations:** 1 College of Forestry, Key Laboratory of State Forestry Administration for Silviculture of the Lower Yellow River, Shandong Agricultural University, Tai’an, China; 2 Center for Forest and Grass Genetic Resources of Shandong Province, Jinan, China

**Keywords:** *Tilia amurensis*, SSR, tree genetics, genetic structure, demographic history

## Abstract

*Tilia amurensis*, a deciduous broad-leaved tree, is distributed in regions neighboring those of *Tilia japonica* populations in China; these plants belong to the Malvaceae family. To date, the genetic status and demographic history of *T. japonica* remain unknown, and the demographic history of *T. amurensis* is not well defined. This study uses standard population genetic and approximate Bayesian computation (ABC) analyses of SSR data to determine the genetic status and divergence times of these two lime taxa. The results revealed that the genetic diversity of *T. japonica* (*I* = 1.181, *H_O_* = 0.484, *H_E_* = 0.602) was high at the population level. The results of the genetic structure revealed that the genetic variation was dominated primarily by within-population variation. Additionally, there was significant genetic differentiation and bidirectional introgression between *T. amurensis* and *T. japonica*. The ABC analysis suggests a (Middle) Pleistocene divergence. These findings have important implications for the formulation of appropriate conservation strategies. Specifically, the clarified divergence time and evidence of genetic exchange indicate that the two species should be protected based on their respective population conditions.

## 1 Introduction

Deforestation and forest fragmentation are the primary threats to forest plants (Lindenmayer and Fischer, 2013; Noh et al., 2022). Owing to the threat posed by various factors (such as human interference, habitat destruction and reproductive challenges), the population of *Tilia* has been declining annually, its distribution range has been shrinking and becoming fragmented. Among them, *Tilia amurensis* has been listed as a Class II Protected Wild Plant in China (Cui and Mu, 2016; Chen et al., 2023; Zhang et al., 2024). The evolutionary potential of a species in nature depends on its level of genetic variation and differentiation. Studies of threatened species can improve the understanding of genetic distribution within and among populations to establish effective strategies for species conservation and management (Hedrick and Miller, 1992).

The earliest *Tilia* fossils were found at the end of the Late Cretaceous and are based on morphological genera established from incomplete leaf fossils, whereas more reliable linden fossils occur in post-Palaeocene strata (Engler, 1909). *Tilia* fossils have been recorded for all periods from the Tertiary to the Pleistocene and are particularly well represented in East Asia (Engler, 1909; Berry, 1918). This suggests that the ancestral genealogy of *T. amurensis* and *Tilia japonica* in China may date back tens of millions of years. With the expectation of continued climate change and further range shifts, habitat fragmentation caused by human disturbance and overexploitation is threatening the survival of *T. amurensis* and *T. japonica* (Logan et al., 2018; Zhang et al., 2024; Mu and Liu, 2007). *T. amurensis*, a deciduous broad-leaved tree, grows mainly in northern China and North Korea, west of the upper reaches of the Yenisei River and east of the Korean Peninsula, where its exhibits an overlapping distribution with the closely related *T. japonica* in the Shandong region of China (Ya and Ren, 1996). There are subtle differences in morphology between *T. amurensis* and *T. japonica* in terms of leaves, bracts and inflorescences, but individuals in the ecotone are difficult to distinguish morphologically (Chen, 1990; Committee, 2007). Consequently, elucidating the spatiotemporal changes across their distribution ranges and the genetic profiles of the closely related species *T. amurensis* and *T. japonica* populations is of significant importance. Previously, we performed a preliminary study of the genetic status of *T. amurensis* and detected possible introgression between *T. amurensis* and *T. japonica* (Wu et al., 2024). However, the genetic background and demographic history of *T. amurensis* and its closely related species have not been thoroughly investigated. Therefore, in this study, we used simple sequence repeat (SSR) markers based on previous studies to determine the genetic structure of natural populations of *T. amurensis* and *T. japonica* in different distribution regions in China and inferred their divergence time from a common ancestor, thereby elucidating the evolutionary history of *T. amurensis* and *T. japonica* and providing a theoretical basis for the effective conservation and rational utilization of *Tilia* genetic resources.

## 2 Materialw and methods

### 2.1 Sample collection and DNA isolation

**Figure 1:**
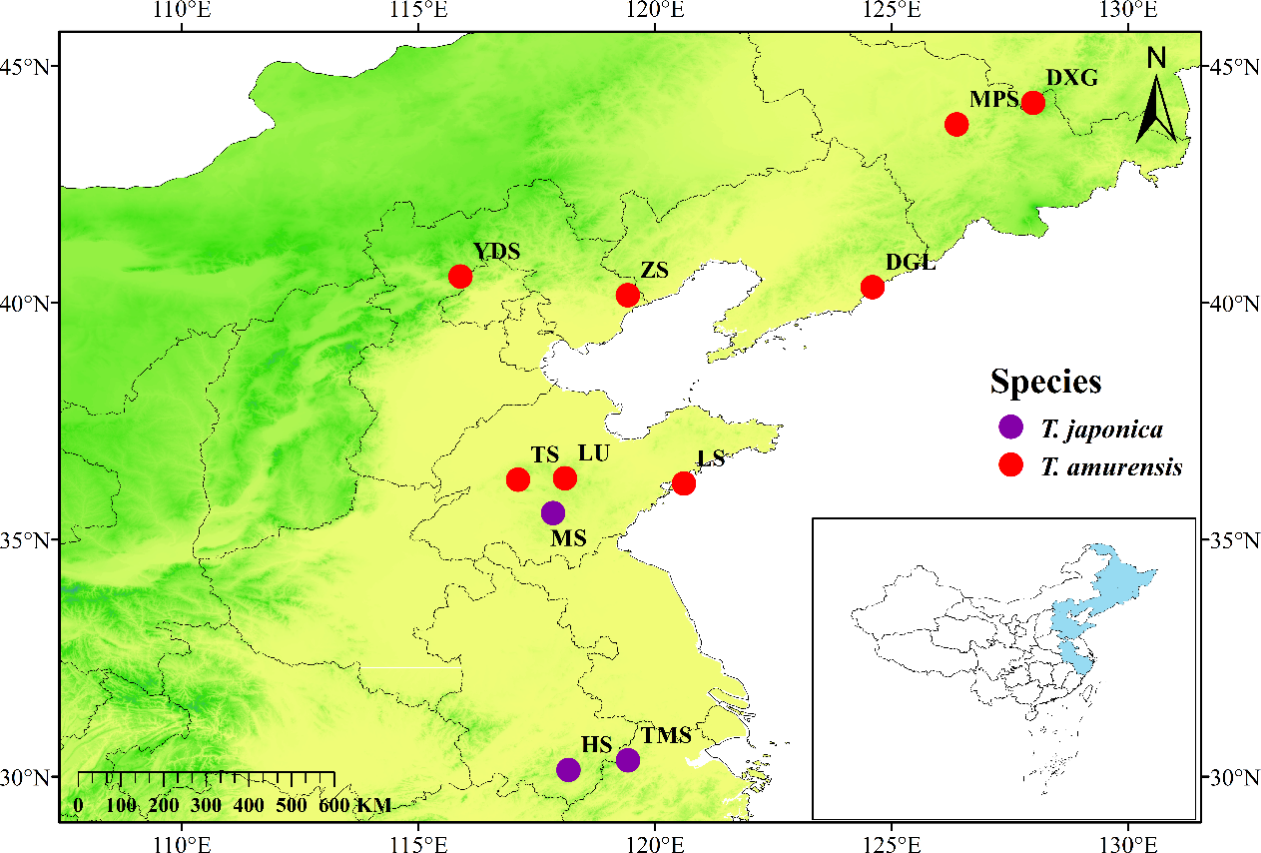
Geographic locations of the populations of *Tilia* included in this study. Descriptions of abbreviations for the study sites can be found in **Table 1**.

The targeted sites were in Heilongjiang, Jilin, Liaoning, Beijing, Hebei, Shandong, Anhui and Zhejiang Provinces in China (four sites in Shandong and one site in each of the remaining provinces). A total of 242 individuals were collected with eight *T. amurensis* populations (TS, LU, LS, ZS, YDS, DGL, MPS, and DXG) and three *T. japonica* populations (HS, MS and TMS) (**Table 1**; **Figure 1**). The sampling work was carried out from May to July 2023. The selection of target plants strictly followed the principle of random sampling, with a minimum distance of 30 meters between any two sampled individuals (the target sample size for each population was 30 plants; if the actual number of individuals was fewer than 30, all were sampled). Fresh young leaves of linden trees were collected, identified by Prof. Dekui Zang, and preserved in silica gel for subsequent DNA extraction. Voucher specimens were also preserved in the herbarium of Shandong Agricultural University.

**Table 1:** Information for the *Tilia* sampling sites.

Population label	Species	Location	Latitude (°N)	Longitude (°E)	Altitude (m)	*N*
TS	*T. amurensis*	Mt. Tai, Taian City, Shandong Prov.	36.263828	117.108972	1422	26
LU	*T. amurensis*	Mt. Lu, Zibo City, Shandong Prov.	36.294079	118.101904	947	26
YDS	*T. amurensis*	Mt. Yudu, Beijing City.	40.553270	115.894394	875	22
ZS	*T. amurensis*	Mt. Zu, Qinhuangdao City, Hebei Prov.	40.158517	119.428285	849	22
LS	*T. amurensis*	Mt. Lao, Qingdao City, Shandong Prov.	36.185469	120.614545	812	29
DGL	*T. amurensis*	Mt. Dagu, Dandong City, Liaoning Prov.	40.330044	124.602425	790	21
MPS	*T. amurensis*	Mt. Mopan, Jilin City, Jilin Prov.	43.763505	126.379051	761	13
DXG	*T. amurensis*	Mt. Daxiagu, Wuchang City, Heilongjiang Prov.	44.218201	127.995354	1136	25
HS	*T. japonica*	Mt. Huang, Huangshan City, Anhui Prov.	30.137615	118.175653	1235	24
MS	*T. japonica*	Mt. Meng, Linyi City, Shandong Prov.	35.559423	117.849874	1221	19
TMS	*T. japonica*	Mt. Tianmu, Hangzhou City, Zhejiang Prov.	30.343229	119.432020	1345	15

TS: population of *T. amurensis* in Mt. Tai; LU: population of *T. amurensis* in Mt. Lu; YDS: population of *T. amurensis* in Mt. Yudu; ZS: population of *T. amurensis* in Mt. Zu; LS: population of *T. amurensis* in Mt. Lao; DGL: population of *T. amurensis* in Mt. Dagu; MPS: population of *T. amurensis* in Mt. Mopan; DXG: population of *T. amurensis* in Mt. Daxiagu; HS: population of *T. japonica* in Mt. Huang; MS: population of *T. japonica* in Mt. Meng; TMS: population of *T. japonica* in Mt. Tianmu; *N*: number of samples.

We used the SteadyPure Plant Genomic DNA Extraction Kit (CTAB method) for DNA extraction, and the experimental procedures were performed according to the methods of Wu’s study (Wu et al., 2024). To evaluate the quality and concentration of the extracted DNA, we used the NanoDrop™ One/OneC Microvolume UV-Vis Spectrophotometer (Thermo Fisher Scientific, USA) for measurement. In accordance with the experimental requirements, the DNA solution was diluted to 30 ng·μl^-1^ for subsequent PCR amplification experiments.

### 2.2 PCR amplification of SSRs

We obtained SSR primers reported for the study of *Tilia* species and selected 27 microsatellite primers with relatively simple repeat motifs and high polymorphism rates for screening. The primers were synthesized by Shanghai Personalbio Technology Co., Ltd. Fluorescent dyes were added to the 5’ end of each primer. The addition of a fluorescent label enhances the functionality, stability, and detection efficiency of the primers, facilitating the rapid identification of amplification products in subsequent high-efficiency capillary electrophoresis. A total of 15 SSR primers from Yue’s study were used for subsequent experiments (**Supplementary Table S1**) (Yue et al., 2022). The reaction system for amplification and the conditions and parameters for PCR were as described in our previous study (Wu et al., 2024). The PCR amplification products were subsequently detected using 1.5% agarose gel electrophoresis. The PCR products that met the experimental requirements were purified and sent to Shanghai Personalbio Technology Co., Ltd., for high-efficiency capillary electrophoresis analysis.

### 2.3 Genetic diversity and population structure

The genetic diversity was estimated on the basis of the SSR data and analyzed using GenAlExv6.51 software to obtain the observed allele number (*Na*), efficient allele number (*Ne*), Shannon’s information index (*I*), observed heterozygosity (*H_O_*), expected heterozygosity (*H_E_*) and inbreeding coefficient (*F_IS_*) (Peakall and Smouse, 2006). The obtained data were subsequently subjected to AMOVA and PCoA via GenAlExv6.51 (Smouse and Peakall, 2012). The genetic differentiation among populations was calculated via the genetic differentiation coefficient (*F_ST_*), and the gene flow between populations was determined using the *F_ST_* value: *N_m_* = (1/*F_ST_* − 1)/4 (Weir and Cockerham, 1984). UPGMA clustering analysis was performed using PopGen32, and a phylogenetic tree was constructed via MEGA7 to determine the genetic relationships among populations (Kumar et al., 2016). We used the Bayesian clustering method in STRUCTUREv.2.3.4 to investigate population structure (Pritchard et al., 2000). The optimal genetic group number K was determined by setting the “length of burn-in period” to 1,000, the “number of MCMC reps after burn-in” to 20,000, and calculating the ΔK value (Evanno et al., 2005).

### 2.4 Demographic history and effective population size

**Figure 2:**
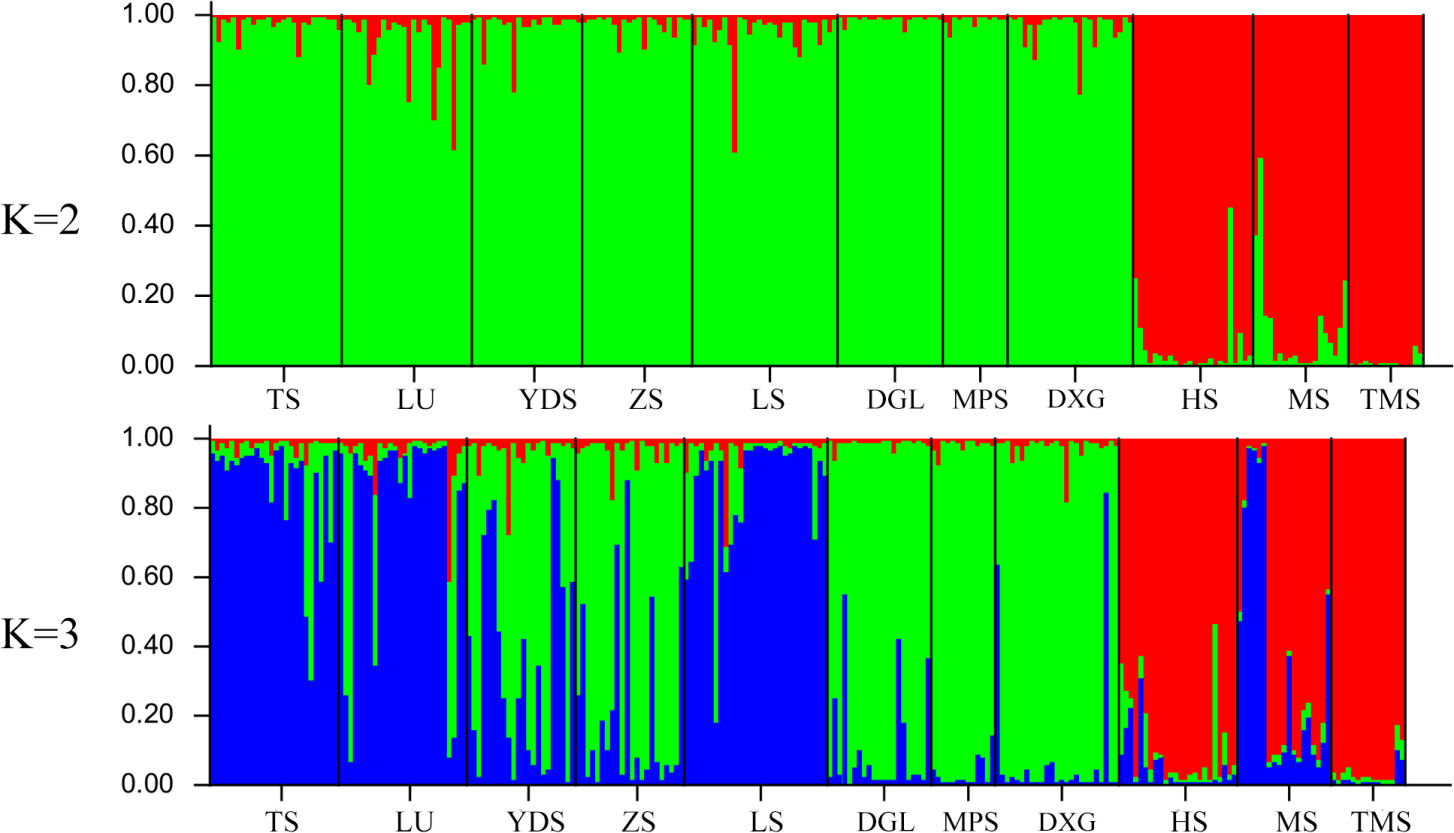
Bar plots for eleven *Tilia* populations at K = 2 and K = 3 according to STRUCTURE analysis. Descriptions of abbreviations for the study sites can be found in **Table 1**.

**Figure 3:**
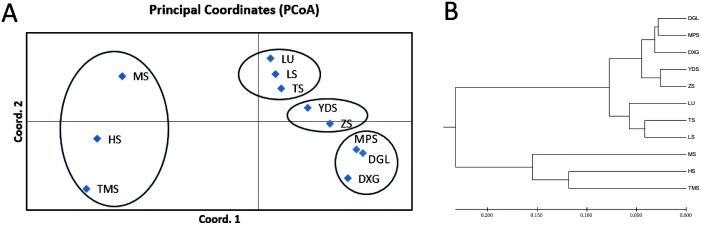
**(A)** Principal coordinate analysis (PCoA) of eleven *Tilia* populations. **(B)** Phylogenetic trees of eleven *Tilia* populations. Descriptions of abbreviations for the study sites can be found in **Table 1**.

**Figure 4:**
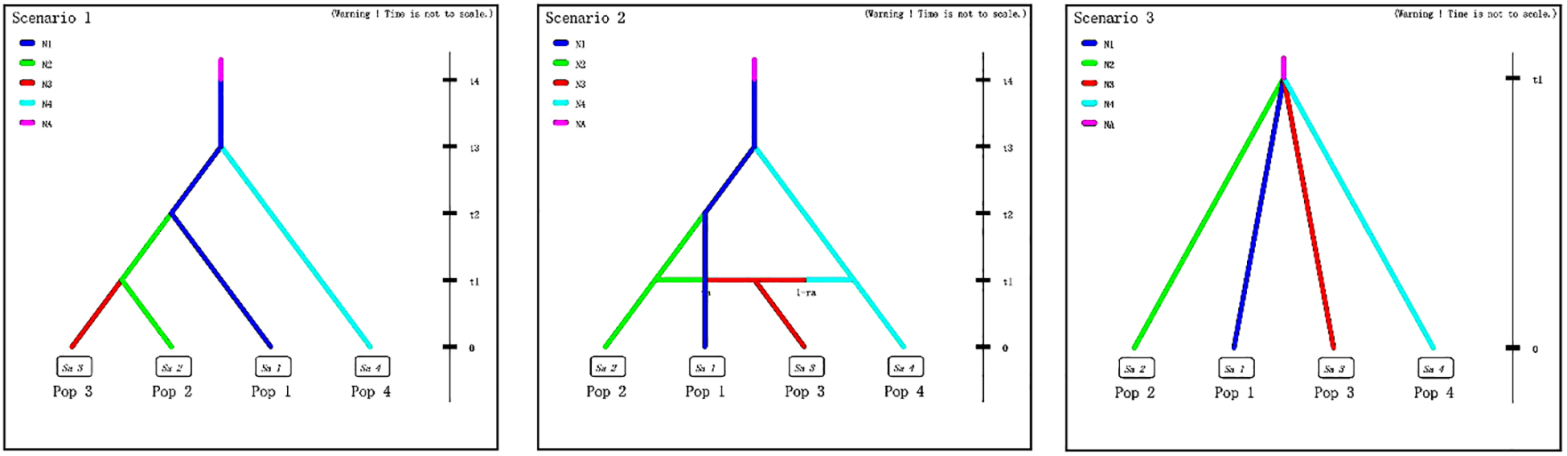
Three scenarios of population group demographic history examined via DIYABC analysis of *T. amurensis* and *T. japonica*; t# is the time scale measured for the generation. N1–4 and NA are the effective population sizes of the corresponding population groups, that is, Pop1 (DGL, MPS and DXG), Pop2 (YDS and ZS), Pop3 (TS, LU and LS), Pop4 (HS, MS and TMS) and the ancestral population.

The approximate Bayesian computation (ABC) method, implemented in DIYABC v 2.1.0, was used to infer the demographic history of *T. amurensis* and *T. japonica* (Cornuet et al., 2014). On the basis of the findings from three approaches (STRUCTURE analysis, phylogenetic tree and PCoA) (**Figures 2**, **3**) and the geographic distribution of the *Tilia* populations (**Figure 1**), four population groups were defined (**Figure 4**). Pop1 was composed of three Northeast China sites (DGL, MPS and DXG), Pop2 was composed of two North China sites (YDS and ZS), Pop3 was composed of three Shandong sites (TS, LU and LS), and Pop4 included the remaining three *T. japonica* sites (HS, MS and TMS). In scenario 1, Pop2 and Pop3 merged at time t1, Pop1 was present at time t2, and Pop4 was present at t3. The ancestors (NA) reached sufficient population size at t4. In scenario 2, Pop3 was assumed to have originated from an admixture of Pop2 and Pop4 at time t1. The rate of admixture of Pop2 and Pop3 was set as “ra”, and that of Pop3 and Pop4 was set as “1-ra”. Pop2 merged with Pop1 at time t2 and finally with Pop4 at time t3. NA reached sufficient population size at t4. In both scenarios 1 and 2, *T. amurensis* and *T. japonica* completed their divergence at time t3. Finally, in scenario 3, the four population groups merged simultaneously at t1. The prior values were set for the effective population size and divergence time estimated with a uniform distribution for all the parameters (**Supplementary Table S2**). The effective population size for each population group (N1, N2, N3, and N4) ranged from 10 to 20,000, with the constraint that all values were greater than or equal to the ancestral population size (NA). The divergence time (td) was set to a minimum of 10 generations and a maximum of 10,000 generations. *Tilia* plants can first flower when they are 12–40 years old, and the trees can live for >450 years (Pigott, 2012).With this in mind, we used a conservative generation time of 100 years. For each scenario, a mutation model was assumed with Mean mutation rate ranging from 10^−4^ to 10^−3^, Individual locus mutation rate ranging from 10^−5^ to 10^−2^, Mean coefficient *P* ranging from 10^−1^ to 3×10^−1^, Individual locus coefficient *P* ranging from 10^−2^ to 9, Mean SNI rate ranging from 10^−8^ to 10^−4^, and Individual locus SNI rate ranging from 10^−9^ to 10^−3^. Single-nucleotide insertion/deletion (SNI) rates were derived from 10,000 simulations generated for each putative scenario. The posterior probabilities were determined following 1,000,000 simulations for each scenario. The goodness-of-fit of the three scenarios was also evaluated by principal component analysis (PCA) using the option “model checking” in DIYABC.

## 3 Results

### 3.1 Genetic diversity

The results of the SSR data analysis revealed the level of genetic diversity in the *T. amurensis* and *T. japonica* samples (**Table 2**). The level of genetic diversity in *T. japonica* was determined from Shannon’s information index (*I*), the observed heterozygosity (*H_O_*), and the expected heterozygosity (*H_E_*). The Shannon’s information index (*I*) of the *T. amurensis* populations varied between 0.889 and 1.231, whereas that of *T. japonica* populations ranged from 0.915 to 1.381. The observed heterozygosity (*H_O_*) of *T. amurensis* populations ranged from 0.329 to 0.647, and the expected heterozygosity (*H_E_*) ranged from 0.440 to 0.588. The observed heterozygosity (*H_O_*) of *T. japonica* populations ranged from 0.426 to 0.606, and the expected heterozygosity (*H_E_*) ranged from 0.520 to 0.657. The fixation indices (*F_IS_*) were positive for all three *T. japonica* populations (**Table 2**).

**Table 2:** Genetic diversity parameters among the three populations of *T. amurensis* and *T. japonica*.

Species	Population	*Na*	*Ne*	*I*	*H_O_*	*H_E_*	*F_IS_*
*T. amurensis*	TS	5.933 ± 0.700	3.229 ± 0.421	1.231 ± 0.154	0.647 ± 0.077	0.588 ± 0.067	-0.127 ± 0.070
	LU	5.400 ± 0.675	2.869 ± 0.329	1.125 ± 0.152	0.534 ± 0.085	0.551 ± 0.071	0.000 ± 0.096
	YDS	6.133 ± 0.894	3.185 ± 0.558	1.166 ± 0.175	0.501 ± 0.091	0.544 ± 0.073	0.088 ± 0.111
	ZS	6.133 ± 0.816	3.174 ± 0.566	1.171 ± 0.173	0.544 ± 0.086	0.537 ± 0.070	-0.004 ± 0.102
	LS	5.933 ± 0.679	2.937 ± 0.425	1.150 ± 0.145	0.494 ± 0.078	0.553 ± 0.062	0.098 ± 0.108
	DGL	4.667 ± 0.504	2.238 ± 0.290	0.899 ± 0.138	0.385 ± 0.078	0.440 ± 0.067	0.195 ± 0.117
	MPS	4.000 ± 0.632	2.347 ± 0.329	0.889 ± 0.155	0.439 ± 0.088	0.452 ± 0.070	0.039 ± 0.120
	DXG	5.533 ± 0.899	2.649 ± 0.571	0.982 ± 0.167	0.329 ± 0.072	0.458 ± 0.065	0.184 ± 0.120
	Mean	5.467 ± 0.261	2.828 ± 0.158	1.077 ± 0.055	0.484 ± 0.029	0.515 ± 0.024	
*T. japonica*	HS	7.067 ± 0.613	3.498 ± 0.522	1.381 ± 0.106	0.516 ± 0.069	0.657 ± 0.032	0.207 ± 0.111
	MS	5.533 ± 0.467	3.010 ± 0.232	1.246 ± 0.093	0.606 ± 0.087	0.630 ± 0.039	0.047 ± 0.130
	TMS	3.267 ± 0.228	2.362 ± 0.232	0.915 ± 0.087	0.426 ± 0.092	0.520 ± 0.044	0.221 ± 0.147
	Mean	5.289 ± 0.352	2.957 ± 0.213	1.181 ± 0.061	0.516 ± 0.048	0.602 ± 0.023	

*Na*: number of observed alleles; *Ne*: effective number of alleles; *I*: Shannon’s information index; *H_O_*: observed heterozygosity; *H_E_*: expected heterozygosity; *F_IS_*: inbreeding coefficient. A description of abbreviations for the study sites can be found in **Table 1**.

### 3.2 Genetic structure

The analysis of molecular variance (AMOVA) was implemented on the basis of SSR data and revealed the molecular variation attributable to differentiation among and within the populations for *T. amurensis* and *T. japonica* (**Table 3**). For *T. amurensis*, 12% of genetic variation exists among populations, while 88% exists within populations. In contrast, for *T. japonica*, 14% of the genetic variation was observed among populations, with the remainder (84%) existing within populations. All the results were highly significant (P < 0.001). The genetic differentiation coefficient (*F_ST_*) among the populations of *T. amurensis* was 0.073, and the gene flow (*N_m_*) was 3.191 (**Table 3**). The *T. japonica* populations, on the other hand, presented relatively high genetic differentiation (*F_ST_* = 0.094) coefficients and relatively low gene flow (*N_m_* = 2.403) (**Table 3**).

**Table 3:** Hierarchical partitioning of genetic variance in *Tilia* using AMOVA.

Species	Source of variation	d.f.	Sum of squares	Estimated variance of components	Percentage of variation (%)	P value^a^
*T. amurensis*	Among populations	7	266.473	1.272	12	<0.001
	Within populations	176	1579.081	8.972	88	<0.001
	Total	183	1845.554	10.244	100	
	Fixation index (*F_ST_*)	0.073				
	Gene flow (*N_m_*)	3.191				
*T. japonica*	Among populations	2	102.081	2.047	14	<0.001
	Within populations	55	669.919	12.180	86	<0.001
	Total	57	772.000	14.227	100	
	Fixation index (*F_ST_*)	0.094				
	Gene flow (*N_m_*)	2.403				

To better understand the relationships between *T. amurensis* and *T. japonica*, the gene flow (*N_m_*) and genetic differentiation coefficient (*F_ST_*) were estimated for all pairs of populations (**Table 4**). The highest gene flow (*N_m_* = 14.568) and smallest genetic differentiation coefficient (*F_ST_* = 0.017) were observed between ZS and YDS. The lowest gene flow (*N_m_* = 0.670) and greatest genetic differentiation coefficient (*F_ST_* = 0.272) were detected between DGL and TMS.

**Table 4:** Estimates of pairwise *N_m_* (above diagonal) and *F_ST_* (below diagonal) values between *Tilia* populations.

Population	TS	LU	YDS	ZS	LS	DGL	MPS	DXG	HS	MS	TMS
TS	0	3.814	4.280	3.877	6.697	2.799	2.298	1.908	1.282	1.567	1.018
LU	0.062	0	5.290	3.372	4.377	1.728	2.017	1.356	1.276	1.751	0.862
YDS	0.055	0.045	0	14.568	4.777	4.123	4.678	3.647	1.275	1.503	0.903
ZS	0.061	0.069	0.017	0	3.091	5.138	5.637	4.687	1.113	1.167	0.806
LS	0.036	0.054	0.050	0.075	0.	2.372	2.356	1.704	1.266	1.791	0.945
DGL	0.082	0.126	0.057	0.046	0.095	0	9.735	7.016	0.809	0.828	0.670
MPS	0.098	0.110	0.051	0.042	0.096	0.025	0	6.771	0.925	0.945	0.690
DXG	0.116	0.156	0.064	0.051	0.128	0.034	0.036	0	0.863	0.843	0.720
HS	0.163	0.164	0.164	0.183	0.165	0.236	0.213	0.225	0	2.986	2.645
MS	0.138	0.125	0.143	0.176	0.123	0.232	0.209	0.229	0.077	0.	1.670
TMS	0.197	0.225	0.217	0.237	0.209	0.272	0.266	0.258	0.086	0.130	0

Descriptions of abbreviations for the study sites can be found in **Table 1**.

**Figure 5:**
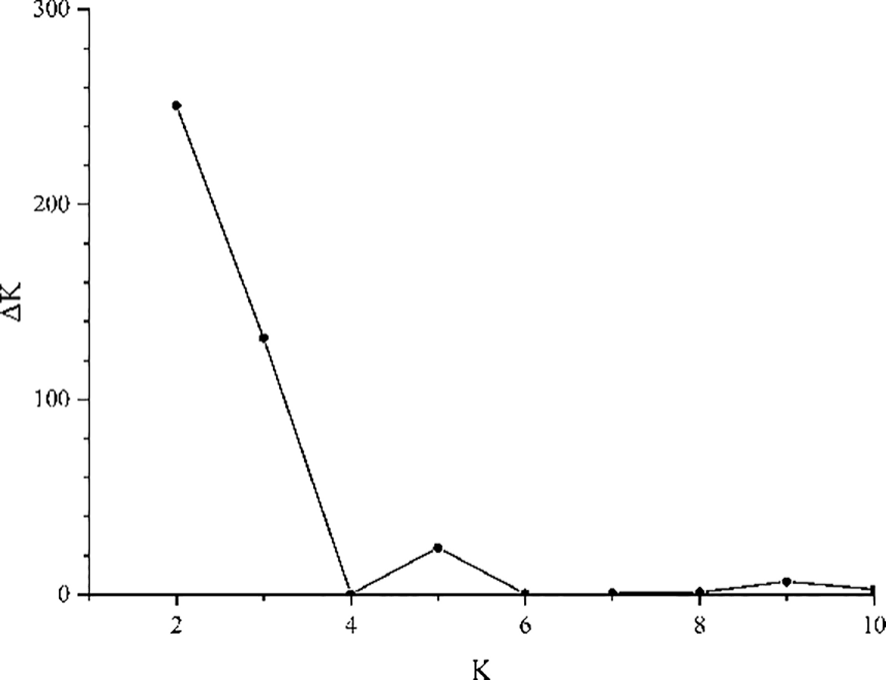
The ΔK value calculated by Structure 2.3.4 shows the maximum at K = 2.

The Bayesian analysis implemented in STRUCTURE revealed a peak at K = 2. The K = 2 model was supported by the highest ΔK value (**Figure 5**), indicating that the most likely number of genetic clusters was 2 (ΔK = 250.88). In contrast, a lower ΔK value was observed at K = 3 (132.47). The bar plot for *Tilia* at K = 2 and 3 is shown in **Figure 2**. At K = 2, the 8 *T. amurensis* populations and the 3 *T. japonica* populations were clearly divided into two groups, with all *T. amurensis* populations categorized in the first group (red) and all *T. japonica* populations categorized in the second group (green). At K = 3, the populations show admixture between different genetic clusters, this being most evident within the MS population.

To validate the results of the population structure analysis, phylogenetic tree were constructed for eleven *Tilia* populations, and PCoA was performed (**Figure 3**). Both analyses showed that populations from TS, LU and LS clustered into one taxon, those from YDS and ZS clustered into one taxon, and those from DGL, MPS and DXG clustered into another taxon. The three *T. japonica* populations (MS, HS and TMS) are clustered together and clearly separated from the *T. amurensis* population. The results were in close agreement with the structural analysis (**Figure 2**).

### 3.3 Demographic history

**Figure 6:**
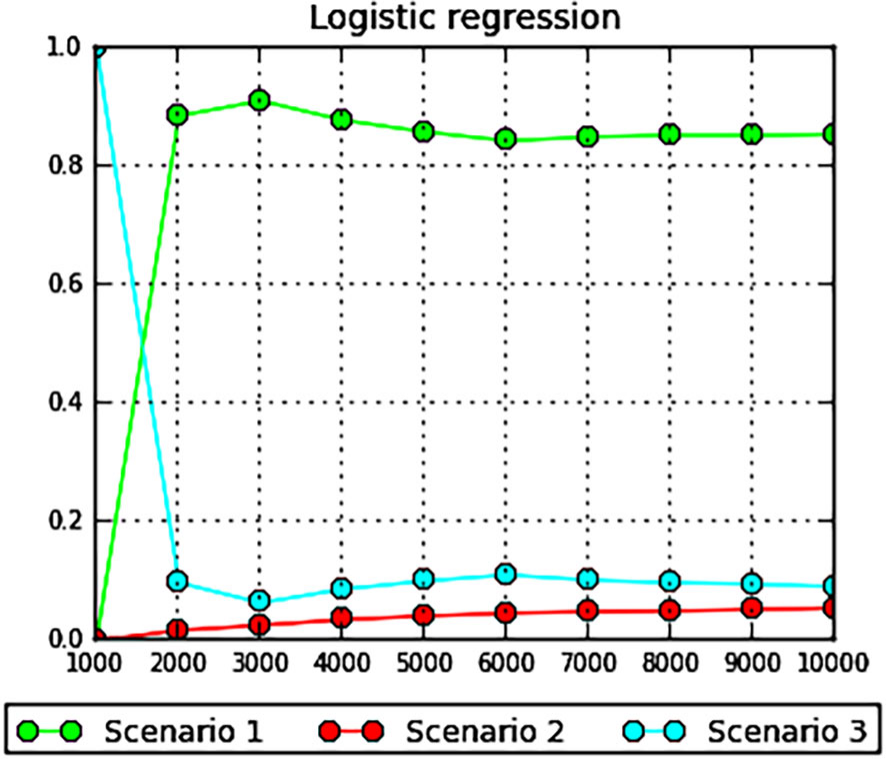
Logistic regression plot for the simulated scenarios in the DIYABC analysis of *Tilia*.

The highest posterior probability after evaluation using the DIYABC analysis of *Tilia* was scenario 1, with p=0.8544 (**Table 5**; **Figure 6**). In Scenario 1, the ancestral population first diverged to give rise to the *T. amurensis* and *T. japonica* groups, with the *T. amurensis* group subsequently differentiating into three distinct population groups. The DIYABC model was evaluated via principal component analysis (PCA) in scenario 1 on the basis of 1000,000 simulations showing the fit between the observed and simulated datasets (**Supplementary Figure S1**). The posterior distribution of each effective population size was also analyzed for the four populations, Pop1, Pop2, Pop3 and Pop4, with median values of 3810, 5840, 9340 and 7610, respectively. The median value of NA was 75.8 for the ancestral population. The mean values of the divergence times t1, t2 and t3 were 287, 416, and 2090 generations (approximately 28.7, 41.6, 209 ka BP), respectively. The time scale of the ancestral population size change, t4, was 4880 generations (approximately 488 ka BP). The prior and posterior distribution patterns are presented in **Supplementary Figure S2**. The mean mutation rates of microsatellites and single-nucleotide insertions/deletions (SNIs) at the examined loci were 8.13×10–^4^ and 8.12×10^-6^, respectively.

**Table 5:** Posterior probability of three putative scenarios in the DIYABC analysis of *Tilia*.

Scenario	1	2	3
Posterior probabilities	0.8544	0.0548	0.0908
Confidence intervals	0.7480-0.9608	0.0185-0.0912	0.0000-0.1888

## 4 Discussion

### 4.1 Genetic diversity

Based on the statistical analysis of hundreds of research papers on genetic variation and population genetic differentiation in different types of plants by Hamrick and Godt (1990), the average expected heterozygosity (*H_O_*) for dicotyledonous plants is 0.136. For long-lived perennial woody plants, the average *H_O_* is 0.177. For plants with an allogamous breeding system and animal-dependent pollination, the average *H_O_* is 0.167. Combined with our previous findings, *T. japonica* (*I* = 1.181, *H_O_* = 0.484, *H_E_* = 0.602) and *T. amurensis* (*I* = 0.982, *H_O_* = 0.516, *H_E_* = 0.515) exhibit comparable levels of genetic diversity, both showing relatively high genetic variation at the population level. It is noteworthy that the conclusions drawn by Hamrick and Godt were based on allozyme data. However, when compared with genetic diversity revealed by molecular markers in species such as the ecologically similar *Picea obovata* (*H_O_* = 0.408; *H_E_* = 0.423) (Kravchenko et al., 2016) and congeneric species including *Tilia sibirica* (*H_E_* = 0.318) (Logan et al., 2018)and *Tilia tomentosa* (*I* = 0.50) (Gabur et al., 2019), *T. japonica* and *T. amurensis* still demonstrate the relatively high level of genetic diversity. According to the statistical analysis by Hamrick and Godt (1990) of hundreds of research papers on genetic variation and population genetic differentiation in different types of plants, the factors influencing the magnitude of genetic variation at the species level are, in order: taxonomic status, distribution range, life form, breeding system, and seed dispersal mechanism. At the population level, the influencing factors are: breeding system, distribution range, life history, taxonomic status, and seed dispersal mechanism. Woody tree species with a wide distribution range, long lifespan, predominantly allogamous breeding system, entomophilous pollination, frequent gene exchange, and abundant seeds that occur in late successional communities exhibit high levels of genetic variation (Hamrick and Godt, 1990). Woody tree species with a wide distribution range, long lifespan, predominantly allogamous breeding system, entomophilous pollination, and frequent gene exchange exhibit high levels of genetic variation (Hamrick and Godt, 1990).

The high genetic diversity of *T. japonica* may be determined by its life history and reproductive system characteristics. Tóth et al. (2022) also suggested that species of the genus *Tilia* are able to maintain a high level of genetic diversity due to their abundance during warmer periods, outcrossing mating system, and increased lifespan. Entomophilous pollination of *Tilia* and the intertwining of intraspecific differentiation and germplasm infiltration phenomena provide opportunities for gene recombination, which leads to high levels of gene flow in *T. japonica* (Ren and Ya, 1995). The reproductive system of *T. japonica*, which is predominantly characterized by sexual reproduction via entomophily, ensures a high level of genetic diversity within its populations (Boo and Park, 2016; Barker et al., 2022). Compared with the narrow distribution ranges of its congeners *T. sibirica* and *T. argentea*, *T. japonica* has a relatively wide distribution in eastern China and Japan. This broader distribution range may have led to higher levels of genetic diversity in *T. japonica* than in other plants of the same genus. Even though the Ne value was lower than the Na value in all *T. japonica* populations, genetic drift did not lead to a reduction in genetic diversity levels. Our results revealed that the genetic diversity of the TMS population was significantly lower than that of the other *T. japonica* populations. The inbreeding coefficients of *T. japonica* populations were greater than 0, and that of the TMS (*F_IS_* = 0.221) population was the largest (**Table 2**), suggesting the possibility of selfing in *T. japonica*. Selfing reduces genetic diversity by decreasing genetic variation and weakening gene flow among populations, which may account for the relatively low genetic diversity observed in TMS populations (Jullien et al., 2019; Clo et al., 2019).

### 4.2 Genetic differentiation and introgression

According to the clear rules of genetic differentiation between populations (*F_ST_* < 0.05, low; 0.05< *F_ST_* < 0.15, medium; *F_ST_ >*0.15, high) defined by Wright (1978) and the results of the current study, there is a moderate degree of genetic differentiation between populations of both *T. amurensis* (*F_ST_* = 0.073) and *T. japonica* (*F_ST_* = 0.094). Moreover, AMOVA revealed that most of the genetic variation was distributed within the populations of *T. amurensis* and *T. japonica*. Genetic differentiation among populations is strongly influenced by gene flow and genetic drift (Andrews, 2010). When *N_m_* > 1, there is sufficient gene flow between populations to exert a homogenizing effect, counteracting genetic drift and preventing differentiation. When *N_m_* > 4, there is high gene flow between populations, and the populations can be regarded as a single unit of random mating (Wright, 1931). Both populations of *T. amurensis* (*N_m_* = 3.191) and *T. japonica* (*N_m_* = 2.403) exhibit strong gene flow, which can reduce local variation and prevent adaptive differentiation, thereby leading to lower genetic differentiation among their populations (Xiang et al., 2015; Wang et al., 2025). According to the study by Hamrick and Loveless (2019), the level of gene flow in plants is significantly influenced by their reproductive systems and seed dispersal mechanisms. Specifically, the mode of plant reproduction (such as selfing or outcrossing) and the mode of seed dispersal (such as wind dispersal or animal dispersal) are key factors in determining the intensity of gene flow. For instance, plants that predominantly rely on outcrossing typically exhibit higher levels of gene flow, whereas selfing plants tend to have relatively lower gene flow (Ballesteros-Mejia et al., 2016; Noreen et al., 2016; Gamba and Muchhala, 2023). *Tilia* species primarily reproduce through outcrossing via insect-mediated pollination, which can be considered the main factor contributing to the gene flow and population structure of populations of *T. amurensis* and *T. japonica*.

The results of the STRUCTURE analysis of 242 individuals revealed that the *T. amurensis* populations and the *T. japonica* populations could be well distinguished when the K value was 2, consistent with the two clustering results revealed by the phylogenetic tree and PCoA analysis. All individuals of *T. amurensis* have genetic components from *T. japonica*, and all individuals of the MS population in *T. japonica*, as well as several individuals of HS and TMS, also experienced genetic infiltration from *T. amurensis*. Relative to the HS and TMS populations, due to geographical proximity to the distribution area of *T. amurensis*, the MS population and *T. amurensis* populations exhibit greater gene flow and a lower degree of differentiation. This may be a factor leading to the MS population experiencing greater more infiltration of genes from *T. amurensis.* When the value of K is 3, all individuals of *T. amurensis* have genetic components from *T. japonica*, and all individuals of *T. japonica* have genetic components from *T. amurensis*. Among them, DGL, MPS, and DXG populations had the lowest degree of gradual infiltration, while easily distinguishable from other *T. amurensis* populations. The main reason is also that DGL, MPS, and DXG populations are geographically furthest away from *T. japonica* distribution area. The presence of significant gene infiltration between the two species implies that the two species may have originated from the same ancestor (Sork, 2016; Ribicoff et al., 2025).

### 4.3 Pleistocene split between *T. amurensis* and *T. japonica*

The ABC analysis in DIYABC revealed that *T. amurensis* and *T. japonica* share the same ancestral origin in this scenario, after which *T. amurensis* diverged into three population groups. Assuming a generation time of 100 years for *Tilia* and considering the summary statistics, the parameter set and the SSR markers used, we can infer a Middle Pleistocene split between *T. amurensis* and *T. japonica*, approximately 2090 generations ago (approximately 209 ka BP). In the Late Pleistocene, the *T. amurensis* population group in Northeast China (DGL, MPS and DXG) diverged from those in North China (ZS and YDS), approximately 416 generations ago (approximately 41.6 ka BP). Additionally, divergence from the Shandong population group (TS, LU and LS) occurred approximately 287 generations ago (approximately 28.7 ka BP). Some of the earliest evidence for putative *Tilia* species dates back to the Tertiary (Wolfe and Wehr, 1987; Pigott, 2012), and the approximate age of *Tilia* was inferred to be 17 million years (Richardson et al., 2015). A recently approximated age for the divergence of *Tilia* was inferred to be approximately 447 ka BP, also during the Middle Pleistocene (Logan et al., 2018). Some studies suggest that climatic fluctuations from the Middle to Late Pleistocene strongly affected tree species, causing lineage divergence. For example, *Quercus* spp diverged during the Early Pleistocene, *Populus alba* and *P. davidiana* diverged in the Middle Pleistocene as they spread southward from the Qinling Mountains, and the North American tree species *Populus balsamifera* and *P. trichocarpa* diverged during the Late Pleistocene at approximately ∼75 ka BP (Levsen et al., 2012; Richardson et al., 2015; Hou and Li, 2022). During the Middle and Late Pleistocene, the pronounced alternation of glacial and interglacial periods and the expansion and contraction of the ice sheet greatly influenced the population dynamics of *Tilia* (Groisman et al., 2013). During the Ice Age, when the climate is cold and dry, the distribution of linden populations shrinks to lower altitudes and latitudes, while during the interglacial period, when the climate warms, *Tilia* populations expand rapidly (Bolikhovskaya and Shunkov, 2014). *T. amurensis* taxa that had been migrating southward since the Miocene were strongly affected in their genetic structure during this repeated expansion and contraction, leading to genetic isolation and differences, which may have subsequently led to a Middle/Late Pleistocene split. The formation of today’s *Tilia* range is the result of redispersal after the end of the most recent ice age (approximately 15 ka BP).

### 4.4 Conservation implications

Both *T. amurensis* and *T. japonica* are vulnerable to habitat loss and overexploitation. Habitat fragmentation caused by anthropogenic disturbances threatens the survival of *Tilia* (Mu and Liu, 2007; Zhang et al., 2024). Currently, there are no effective conservation measures in place for *T. amurensis* and *T. japonica*. Our results indicate that the level of genetic diversity is high in *T. japonica* and that genetic variation occurs mainly within populations of *T. amurensis* and *T. japonica*. Therefore, *in situ* conservation of these populations is the most effective strategy to preserve their species diversity. The HS and MS populations of *T. japonica* maintain high genetic diversity and should be prioritized for *in situ* conservation. The complete biological conservation strategy should combine *in situ* and translocated conservation (Volis and Blecher, 2010; Dulloo et al., 2010; Duman et al., 2024). Therefore, to avoid inbreeding decline and population degradation, relocation protection and seed collection should also be strengthened. The shared ancestral background and the presence of bidirectional gene introgression between *T. amurensis* and *T. japonica* suggest that genetic connectivity between these two species should be considered in their conservation and management. To prevent population degradation and loss of genetic resources due to excessive hybridization between the two species, the protection of the three *T. amurensis* populations in Northeast China (DGL, MPS and DXG) and the TMS population of *T. japonica*, which have a purer genetic structure with less genetic drift, should be strengthened. Furthermore, conserving the genetic diversity of these two species requires attention not only to their respective core distribution areas but also to their zones of distributional overlap, which represent critical regions for gene flow.

## 5 Conclusion

We observed significant genetic differences and bidirectional gene infiltration through genetic studies of *T. amurensis* and *T. japonica*. Considering a generation time of 100 years, we estimate by DIYABC that the splitting of *T. amurensis* and *T. japonica* likely occurred in the Middle Pleistocene. The differentiation of the three *T. amurensis* population groups also occurred in the Late Pleistocene. Combined with the results of previous studies, our findings revealed that *T. amurensis* and *T. japonica* presented high levels of genetic diversity. However, given that both *T. amurensis* and *T. japonica* are facing dual pressures of habitat loss and overexploitation, we still strongly recommend a focus on conservation work for both species. The natural distribution areas of *T. amurensis* and *T. japonica* should be restored, and protection and management of these species should continue.

## Data Availability

The raw data supporting the conclusions of this article will be made available by the authors, without undue reservation.
