# Supplementary figures and images for: Analysis of the population genetic structure and demographic history of Tilia amurensis and Tilia japonica in China using SSR markers
Source: Front Plant Sci. 2025 Dec 11;16:1651814. doi: 10.3389/fpls.2025.1651814 (PMC12738890; doi:10.3389/fpls.2025.1651814)

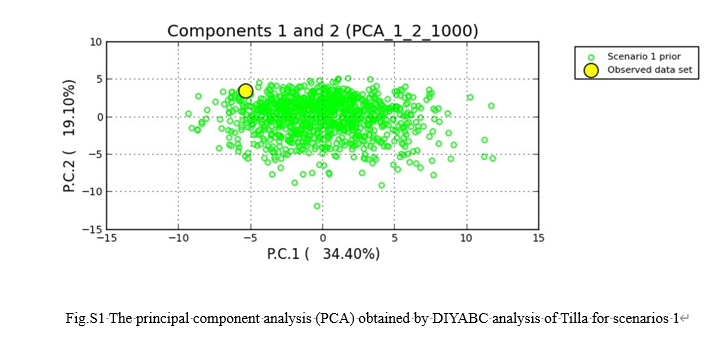

Supplement: Supplementary file 1 [file Image1.tif]

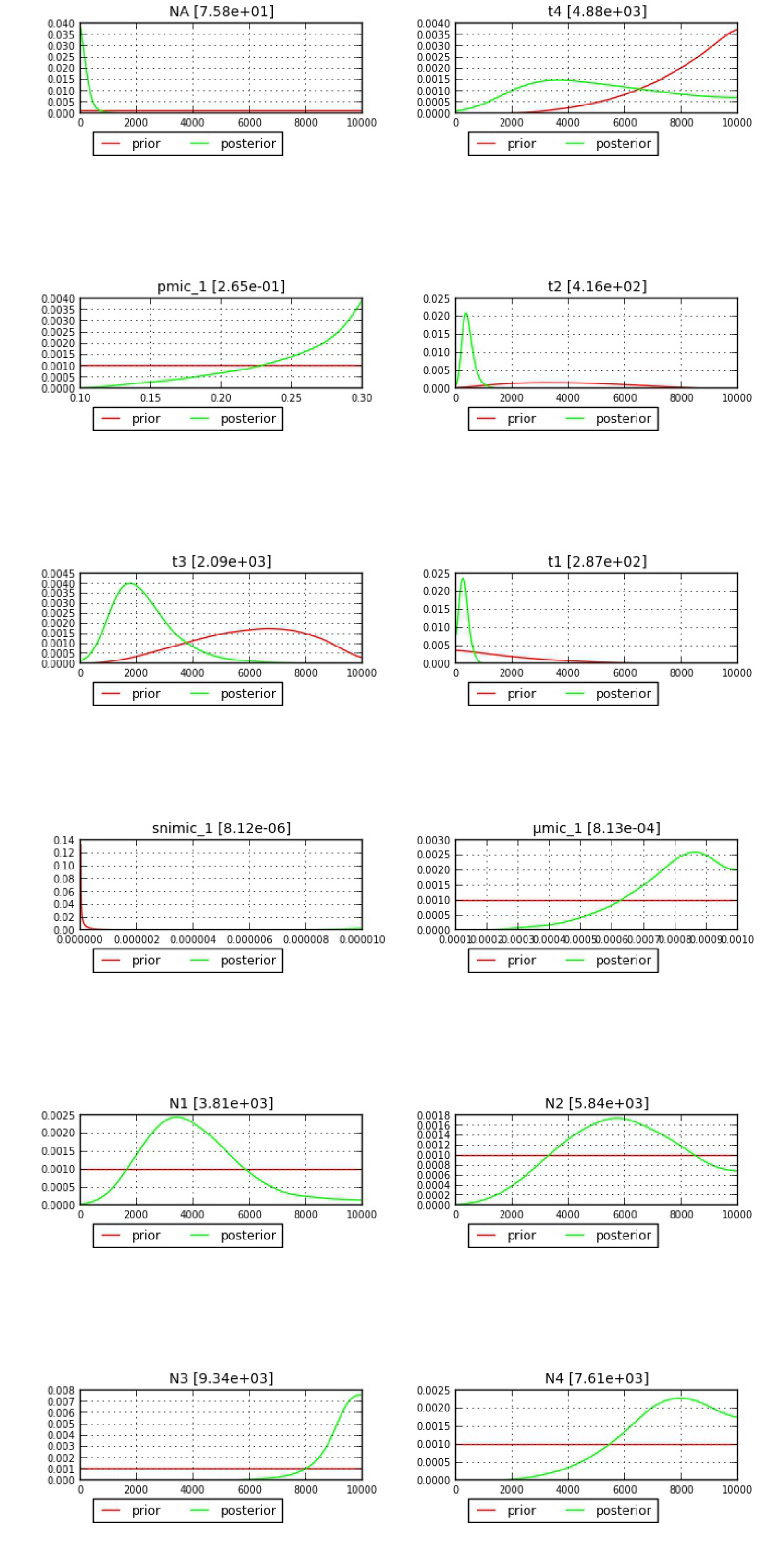

Supplement: Supplementary file 2 [file Image2.tif]
