# Supplementary material for: Analysis of the population genetic structure and demographic history of Tilia amurensis and Tilia japonica in China using SSR markers
Source: Front Plant Sci. 2025 Dec 11;16:1651814. doi: 10.3389/fpls.2025.1651814 (PMC12738890; doi:10.3389/fpls.2025.1651814)
Supplement: Supplementary file 3 [file Table1.docx]

**Table S1** The infoemation of primers used in the study

| Primer name | Primer sequence (5'-3') | Observed number of alleles | Polymorphic information content (PIC) | Length | GC (%) |
| --- | --- | --- | --- | --- | --- |
| B2405 | TCTGCAAAGGAACTCTTCATCA | 6 | 0.7913 | 22 | 40.9 |
|  | TGGATTGCTAGTTGTCAAGCAT |  |  | 22 | 40.9 |
| B2975 | ATTCATCATCGCCCACAGTT | 4 | 0.6414 | 20 | 45 |
|  | TGCTCCACTCGAAAACATGA |  |  | 20 | 45 |
| B2410 | TATAGATGCCCGTTCCAAGC | 6 | 0.7913 | 20 | 50 |
|  | CATCAAGACAAGGGCAATGA |  |  | 20 | 45 |
| B1045 | ACGGCTCCCTTATCACACTG | 4 | 0.6847 | 20 | 55 |
|  | TGCCATCTCACGTAGCTTTG |  |  | 20 | 50 |
| B595 | GGGGCAAGTCCTTCTCTTCT | 7 | 0.8397 | 20 | 55 |
|  | ATGGTGCCCATGACTTTAGC |  |  | 20 | 50 |
| B90 | AGAGCAGAGCCGTTGGATAA | 6 | 0.7913 | 20 | 50 |
|  | CGGTACCACCTAGCTTCGTC |  |  | 20 | 60 |
| B2915 | CGACCTGGTTCTCTGGTTTC | 2 | 0.3698 | 20 | 55 |
|  | CGACCAGGTGCATATAGCCT |  |  | 20 | 55 |
| B505 | CTTCCCCAGCTACCAGTCAG | 3 | 0.5798 | 20 | 60 |
|  | GAAAGGCCAAAGAGACCACA |  |  | 20 | 50 |
| C2155 | CGGTGTGTCGACATTGTTTC | 6 | 0.7913 | 20 | 50 |
|  | CTCTGGAAGAAGGGAACACG |  |  | 20 | 55 |
| C3380 | TTGCATCAACAACAACAGCA | 5 | 0.7397 | 20 | 40 |
|  | CCCAGGCTCATACACTGGTT |  |  | 20 | 55 |
| C280 | CCTGTCCCTAAGCGTCAAAG | 7 | 0.8571 | 20 | 55 |
|  | CTGGGATAGTCAAGGCCTCA |  |  | 20 | 55 |
| C1050 | AACCTGGAGGCTCTGGACTT | 5 | 0.7347 | 20 | 55 |
|  | ACACAGAGGTTCGGAAATCG |  |  | 20 | 50 |
| C110 | AGAGCTGCCTTCAAGCTACG | 4 | 0.7347 | 20 | 55 |
|  | ATACCCCCGGCGTTATACTT |  |  | 20 | 50 |
| D150 | GCTTGTTTTCAAGAGTCGGC | 4 | 0.6939 | 20 | 50 |
|  | ATGGGGAAAATTGTATGGCA |  |  | 20 | 40 |
| E5 | TTGCTGTTTCATTCTGCTGG | 6 | 0.8163 | 20 | 45 |
|  | AGTTTCGAATAGCGCCTTGA |  |  | 20 | 45 |
