# Supplementary material for: Analysis of the population genetic structure and demographic history of Tilia amurensis and Tilia japonica in China using SSR markers
Source: Front Plant Sci. 2025 Dec 11;16:1651814. doi: 10.3389/fpls.2025.1651814 (PMC12738890; doi:10.3389/fpls.2025.1651814)
Supplement: Supplementary file 4 [file Table2.doc]

**Table S2** Prior distributions of the parameters for simulated scenarios in DIYABC analysis^1^ of *Tilia*. A uniform distribution was applied for all parameters. *N*1, *N*2, *N*3 *N*4 and *NA* is effective population sizes of the corresponding populations Pop1, Pop2, Pop3, Pop4 and the ancestral population

| Parameter | Minimum | Maximum |
| --- | --- | --- |
| Effective population size |  |  |
| *N*1 | 10 | 20000 |
| *N*2 | 10 | 20000 |
| *N*3 | 10 | 20000 |
| Time scale in generations |  |  |
| *t*1 | 1 | 10000 |
| *t*2 | 1 | 10000 |
| *t*3 | 1 | 10000 |
| *NA* | 10 | 10000 |
| Admixture |  |  |
| ra | 0.001 | 0.999 |
| Mutation model |  |  |
| Mean mutation rate | 1.00e-004 | 1.00e-003 |
| Individual locus mutation rate | 1.00e-005 | 1.00e-002 |
| Mean coefficient *P* | 1.00e-001 | 3.00e-001 |
| Individual locus coefficient *P* | 1.00e-002 | 9.00e001 |
| Mean SNI rate | 1.00e-008 | 1.00e-004 |
| Individual locus SNI rate | 1.00e-009 | 1.00e-003 |

^1^Cornuet JM, Pudlo P, Veyssier J et al. (2014) DIYABC v.2.0: a software to make approximate Bayesian computation inferences about population history using single nucleotide polymorphism, DNA sequence and microsatellite data. Bioinformatics 30:1187-1189. doi: 10.1093/bioinformatics/btt763
